# The Prevalence of Subjective Symptoms after Exposure to Arsenic in Drinking Water in Inner Mongolia, China

**DOI:** 10.2188/jea.13.211

**Published:** 2007-11-30

**Authors:** Xiaojuan Guo, Yoshihisa Fujino, Jinshan Chai, Kegong Wu, Yajuan Xia, Yanhong Li, Jiangang Lv, Zhiming Sun, Takesumi Yoshimura

**Affiliations:** 1 Inner Mongolia Center for Endemic Disease Control and Research.; 2 Department of Clinical Epidemiology, Institute of Industrial Ecological Sciences, University of Occupational and Environmental Health, Japan.; 3 Station for hygiene prevention of Wuyuan County in Inner Mongolia.

**Keywords:** Arsenic, drinking water, Inner Mongolia, subjective symptoms

## Abstract

Background: In Inner Mongolia, China, more than 300,000 people are chronically exposed to arsenic via their drinking water. We have previously reported that the prevalence of arsenical dermatosis was as high as 40% in the Hetao Plain area. However, the association between exposure to arsenic in drinking water and adverse health effects has not been fully examined. The purpose of this study was to examine the association between exposure to arsenic and prevalence of subjective symptoms.

Methods: A cross-sectional study was carried out in 431 residents of an arsenic-affected village and 189 residents of an arsenic-free village in 1996. Health-related interviews and physical examinations were conducted. The odds ratio for each subjective symptom was estimated, comparing residents of arsenic-free and affected villages.

Results: An arsenic level of 50+ *μ*g/L was found in 90.6% of wells in the arsenic-affected village. Adjusted odds ratios of subjective symptoms, including coughs (odds ratio [OR]=12.8, 95% confidence interval [CI]: 6.4-25.6), stomachaches (OR=5.8, 95% CI: 3.6-9.4), palpitations (OR=3.6, 95% CI: 1.5-8.2), urination problems (OR=14.7, 95% CI: 3.3-65.5) and spontaneous abortions (OR=2.7, 95% CI: 0.8-8.4), were markedly higher amongst residents of the arsenic-affected village, including those without arsenic dermatosis.

Conclusions: The present study shows a high prevalence of subjective symptoms amongst residents of an arsenic-affected village. Symptoms occurred in people with and without arsenic dermatosis. Our findings suggest that symptoms other than dermatosis should be considered when a clinical diagnosis of arsenic toxicosis is made.

Arsenic is a ubiquitous element that is widely distributed in nature. However, in recent years, incidents of arsenic contamination of drinking water have been discovered all over the world. Arsenic contamination in Inner Mongolia was first reported in the 1990s. More than 300,000 people are chronically exposed to drinking water highly contaminated with arsenic.^1^ Most of the arsenic-affected villages are located on the Hetao Plain on the northern side of the Yellow River in Inner Mongolia, China.

Chronic ingestion of inorganic arsenic is known to cause adverse health effects. The most common health problem is dermatosis. There have also been reports indicating that arsenic exposure is associated with cancer and affects various internal organs, such as the circulatory and nervous systems.^2^ Recently, a study from India showed a high prevalence of complaints of cough and fatigue amongst residents of arsenic polluted areas.^3^ There was also a significant association between ingested arsenic levels and prevalence of coughs. However, to date there is little epidemiologic data on other subjective symptoms such as stomachaches, palpitations, insomnia, urination problems.

We have previously reported that the prevalence of arsenical dermatosis was as high as 40% in the Hetao Plain area of Inner Mongolia.^4^ However, the association between exposure to arsenic in drinking water and the occurrence of adverse health effects has not been fully examined. To investigate the association between exposure to arsenic in drinking water and subjective symptoms, a cross-sectional study was carried out in Wuyuan County in Inner Mongolia, China in 1996.

## METHODS

### Study area and population

Based on a preliminary survey conducted between 1993 and 1995, there were 66 arsenic-affected villages (arsenic level of tube well water 50+ *μ*g/L) out of 679 villages in Wuyuan County, while the permitted standard of arsenic in drinking water is 50 *μ*g/L in China. The study area includes an arsenic-affected village and an arsenic-free village in a township of Wuyuan County located in the centre of Hetao Plain. The township studied was the most seriously arsenic-affected region, with a maximum arsenic concentration in wells of 590 *μ*g/L.^5^ In this area there is no industry or mining and the main occupation is agriculture. All irrigation water is drawn from the Yellow River. In the arsenic-affected village four public open wells (depth approximately 3-5 m) were used for drinking water until the 1970s, but since 1980 most of the villagers have owned and used private tubule-type wells (depth approximately 15-30 m).

The arsenic-affected village consists of thirteen districts, and three neighboring districts among them were selected for the present study. Amongst the 649 residents of the arsenic-affected districts, 431 (66.6%, 207 men and 224 women) participated in detailed interviews. Almost all of them were engaged in agricultural work. In the arsenic-free village, 96% of people (90 men and 99 women out of a total of 196 residents) participated in the study. All the subjects who participated in the study provided informed consent.

### Analyses of arsenic levels in well water

A total of 106 samples were collected from households using drinking water wells in the arsenic-affected village and 19 samples in the non-arsenic-affected village. Water samples were analyzed to assess the total arsenic concentration using Ag-DDC analysis at the Institute for Water Research in BAYANNAOER League, Inner Mongolia.

### Interviews and physical examinations

Questions included sociodemographic factors, lifestyle choices (e.g. use of tobacco, alcohol consumption), medical history, areas of residence, occupational history, working conditions and source of household water at their current residence and duration of use.

A careful skin examination was performed by a dermatologist in order to reduce the observation bias. Arsenical deimatosis was diagnosed according to an arsenical poisoning clinical diagnosis standard of Inner Mongolia described in 1994.^6^ The criteria were as follows: (1) a history of residence in an area with a high arsenic concentration or a history of drinking arsenic concentrated water, (2) keratosis on the palms of the hands and/or soles of the feet, and (3) pigmentation and leucomelanosis on the body. A diagnosis was made when criterion (1) was fulfilled along with criterion (2) and/or (3).

Participants were then asked whether they had any health problems or subjective symptoms by a physician interviewer. The following questions were asked: “Do you have problems with chronic coughing?”, “Do you suffer from frequent stomachaches?”, “Do you suffer from palpitations?”, “Are you having trouble urinating (e.g. frequent urination, pain when or after urination)?”, “Do you have arthralgia?” The interviewer also asked women about their history of childbirth and/or spontaneous abortion.

### Statistical analysis

Subjects were classified into three groups: residents in the arsenic-free village, residents in the arsenic-affected village with dermatosis and residents in the arsenic-affected village without dermatosis. Person who currently smoked and daily drank was defined as a current smoker and habitual drinker, respectively. Logistic regression analyses were performed to estimate the prevalence odds ratio (OR) of each subjective symptom, controlling for sex, age, smoking and alcohol consumption, comparing the three groups. Analyses were performed using StatView^®^ (Version 5.0).

## RESULTS

In the arsenic-free village, the mean arsenic concentration of wells was 9.6 (standard deviation [SD] =12.8) *μ*g/L, and the maximum recorded concentration was 43 *μ*g/L. In the arsenic-affected village, 96 of 106 (90.6%) wells contained water in which the arsenic concentration was 50+ *μ*g/L. The maximum recorded concentration was 1354 *μ*g/L. In the arsenic-affected village, the average duration of using tube well water was 12 years (SD=3.22), and the shortest duration was 4 years.

Table 1 shows the characteristics of the subjects who took part in the present study. The age and sex ratio were similar in both groups. The prevalence of arsenic dermatosis was 45.5% in the arsenic-affected village.

**Table 1.** Characteristics of the subjects by residential area.

	Arsenic-free village		Arsenic-affected village	
	
	n=189		n=431	
Mean age, years (SD)	33.5	(16.6)	32.6	(18.9)

Male sex (%)	90	(47.6)	207	(48.0)
Current smoking (%)	54	(28.6)	184	(42.7)
Habitual drinking (%)	49	(25.9)	99	(23.0)
Arsenic dermatosis (%)	*		196	(45.5)

* dermatologic examination was not conducted.

Table 2 shows the prevalence of subjective symptoms in subjects divided according to their residential area. Most symptoms were more common in the arsenic-affected village compared with the arsenic-free village. An exception to this was arthralgia, which occurred at similar levels in the two areas.

**Table 2.** Prevalence of subjective symptoms by the subjects in the arsenic-affected and the arsenic-free village, Inner Mongolia.

	Arsenic-free village		Arsenic-affected village	
	n=189		n=431	
	
	No. of case	%	No. of case	%
Cough	10	5.3	151	35.0
Stomachache	24	12.7	189	43.9
Palpitation	7	3.7	51	11.8
Urination trouble	2	1.1	37	8.6
spontaneous abortion	4	4.0*	24	10.7*
Arthralgia	78	41.2	170	39.4

* Percentage is based on 99 and 224 female subjects in the arsenic-free and the arsenic-affected village

Table 3 shows the multivariate ORs of subjective symptoms in the arsenic-free versus the arsenic-affected villages. Subjects with and without arsenic dermatosis were also compared. In the arsenic-affected village there was a significantly higher odds ratio of coughs (OR=12.8, 95% confidence interval [CI]: 6.4-25.6), stomachaches (OR=5.8, 95% CI: 3.6-9.4), palpitations (OR=3.6, 95% CI: 1.5-8.2), and urinary problems (OR=14.7, 95% CI: 3.3-65.3) than in the arsenic-free village. The OR of spontaneous abortion in the arsenic-affected village was higher than in the arsenic-free village (OR=2.7, 95% CI: 0.8-8.4), and was significantly higher amongst subjects with arsenic dermatosis (OR=3.8, 95% CI: 1.2-12.7) compared to those of the arsenic-free village.

**Table 3.** Multivariate adjusted odds ratiosa (OR) of subjective symptom comparing between the arsenic-affected village and the arsenic-free village.

	All participants of the arsenic-affected village		Without arsenic dermatosis		With arsenic dermatosis	
	n=431		n=235		n=196	
		
	OR^a^	95% CI	OR^a^	95% CI	OR^a^	95% CI
Cough	12.8	6.4 - 25.6	12.3	5.8 - 25.9	13.2	6.4 - 27.1
Stomachache	5.8	3.6 - 9.4	4.8	2.8 - 8.2	6.9	4.0 - 11.7
Palpitation	3.6	1.5 - 8.2	2.2	0.8 - 5.7	4.8	2.1 - 11.5
Urination trouble	14.7	3.3 - 65.5	15.7	3.3 - 75.7	14.0	3.0 - 64.7
Spontaneous abortion	2.7	0.8 - 8.4	1.7	0.5 - 6.1	3.8	1.2 - 12.7
Arthralgia	1.0	0.7 - 1.6	0.9	0.5 - 1.4	1.3	0.8 - 1.9

^a^ ORs adjusted for sex, age, smoking and alcohol consumption, with a reference group of 189 subjects in the arsenic-free village

CI: confidence interval

## DISCUSSION

The present study has examined the prevalence of subjective symptoms in a population living in an arsenic-affected area in Inner Mongolia. In this area 90.6% of wells have water with an arsenic concentration higher than 50 *μ*g/L. There has been very few information on the subjective symptoms related to chronic arsenic exposure, except for respiratory symptoms. Therefore, the general subjective symptoms were selected to examine in the present study, based on the previous studies which reported arsenic induced health problems, such as respiratory injury, gastrointestinal injury, renal injury and any cite of cancers.^7^^-^^9^ The study showed a positive association between chronic exposure to arsenic and the prevalence of coughs, stomachaches, palpitations, urination problems and spontaneous abortions, comparing between the arsenic-affected and the arsenic-free village, even after accounting for sex, age, smoking and alcohol consumption. In addition, in the present study, cough, stomachache, palpitation, and abortion were more likely to prevalent among the subjects with arsenic dermatosis than the subjects without arsenic dermatosis. It is known that skin abnormalities are a hallmark of chronic arsenic poisoning. Furthermore, the skin is sensitive to arsenic’s toxic effects, since the skin localizes and stores arsenic because of its high keratin content.^10^ Thus it may be possible that subjects with arsenic dermatosis may be more susceptible to arsenic perhaps because of the individual patterns of arsenic metabolism, such as the activity of methylating enzymes, or because the metabolite products play an important role in the involvement of arsenic injury.

Long-term ingested inorganic arsenic has been reported to cause damage to various organs,^3^^,^^11^ Effects on the cardiovascular, pulmonary and digestive system were also observed in children and young adults consuming arsenic-contaminated water (mean concentration 600 *μ*g/L) in northern Chile.^12^ In general, skin lesions including keratosis and hyperpigmentation were taken to be the hallmark of chronic arsenic toxicosis and have become a guideline for diagnosis in many countries. However, the present findings suggest that subjective symptoms may be a sub-clinical sign of exposure to arsenic. Thus, symptoms other than dermatosis should be considered when making a clinical diagnosis of arsenic toxicosis.

The present study was a cross-sectional study, so information biases may have occurred. For example, a person who knows his residential area is affected by arsenic may be more likely to report subjective symptoms. However, this is unlikely to have affected our results, since at the time of our investigation most people did not know that there was a problem with arsenic poisoning in their area of residence. Other possible bias is the deference of participation rate between arsenic-affected and arsenic-free village. Sixty-six percent people of the arsenic-affected village participated to the present study, while 96% people of the arsenic-free village did. It is possible that if the person who had or felt health problems likely to participate in the study among the arsenic-affected village, the ORs for subjective symptoms were relatively inflated. However, the most reason of this participation gap between both villages is due to the population size of the villages. The arsenic-free village is a small one, and villagers were likely to access to the study, because they were well announced or easily came to the place where the examination and interviews were held. A further limitation of the present study is the lack of analyses at an individual level as to the degree of arsenic exposure. Although the duration of well use and arsenic level of well water were examined, the accuracy of one-spot evaluation of well water arsenic may be in doubt, since there may be a seasonal variation in the arsenic content of tube-well water. Irrigation is one of many environmental factors that could plausibly affect the arsenic level of underground water. Therefore, it is impossible to correlate the level of arsenic-related symptoms and the dose of arsenic ingested.

The detailed mechanisms by which chronic arsenic exposure induces various subjective symptoms are unclear; however, previous studies have made some suggestions. Inorganic arsenic is readily absorbed after ingestion and is widely distributed around the human body. The hazards of arsenic do not show any unique organotropism.^13^ In Mexico, the prevalence of non-specific symptoms, such as nausea, abdominal pain, and diarrhea, was significantly higher in the population exposed to arsenic.^7^ In Bangladesh, a higher prevalence of coughs was found amongst people in an arsenic-affected area.^8^ It has been also reported that long-term exposure to arsenic induces renal injury including hematuria, leukocyturia, and glycosuria.^9^

The present study indicated that there was a high OR of subjective symptoms including cough, stomachache, palpitations, urination problems and spontaneous abortion amongst residents in the arsenic-affected village. There was even a high OR in inhabitants of the arsenic-affected village who did not have arsenical dermatosis. The findings suggest that symptoms other than dermatosis should be considered when clinically diagnosing arsenic toxicosis.
